# Listening Until the End: Best Practices and Guidelines for Auditory Care in Palliative Sedation in Europe

**DOI:** 10.3390/healthcare13141664

**Published:** 2025-07-10

**Authors:** Ismael Rodríguez-Castellanos, María Isabel Ortega González-Gallego, Alberto Bermejo-Cantarero, Raúl Expósito-González, Julián Rodríguez-Almagro, Sandra Martínez-Rodríguez, Andrés Redondo-Tébar

**Affiliations:** 1 Facultad de Enfermería de Ciudad Real, Universidad de Castilla-La Mancha, 13071 Ciudad Real, Spain; ismael.rodriguez3@alu.uclm.es (I.R.-C.); mariaisabel.ortega@uclm.es (M.I.O.G.-G.); raul.egonzalez@uclm.es (R.E.-G.); julianj.rodriguez@uclm.es (J.R.-A.); sandra.martinez@uclm.es (S.M.-R.); andres.redondo@uclm.es (A.R.-T.); 2 Gerencia de Urgencias, Emergencias y Transporte Sanitario SESCAM, Castilla-La Mancha, 13500 Puertollano, Spain; 3 Centro de Estudios SocioSanitarios, Universidad de Castilla-La Mancha, 16071 Cuenca, Spain

**Keywords:** palliative sedation, end-of-life care, humanized care, hearing preservation, nursing

## Abstract

**Background/Objectives**: Auditory capacity plays a fundamental role in human emotional development from prenatal stages and persists as the last sensory modality to fade during terminal phases. In palliative sedation, uncertainty about preserved hearing—despite potential unconsciousness—underscores the need to evaluate current care recommendations for this critical sensory dimension. This review examines European guidelines to (i) assess auditory care integration in palliative sedation protocols and (ii) propose humanization strategies for sensory-preserving end-of-life care. **Methods**: Narrative review of evidence from the European Palliative Sedation Repository and the European Association for Palliative Care (EAPC). **Results**: Three key findings emerged: (i) lack of explicit protocols for auditory care despite acknowledging environmental sound management (e.g., music, family communication); (ii) limited consensus exists regarding hearing preservation during unconsciousness. **Conclusions**: Although auditory perception during palliative sedation remains scientifically uncertain, the precautionary principle warrants integrating auditory care into palliative sedation through (i) family education on potential hearing preservation; (ii) therapeutic sound protocols; and (iii) staff training in sensory-inclusive practices. This approach addresses current gaps in the guidelines while enhancing patient dignity and family support during end-of-life care. Further research should clarify auditory perception thresholds during sedation.

## 1. Introduction

Palliative care is a critical global health priority, with the World Health Organization (WHO) estimating that 40 million people annually require these services—a number projected to rise due to aging populations and the growing burden of non-communicable diseases [1]. For patients in the last days of life phase, palliative care focuses on ensuring a dignified death, free from preventable suffering, through interventions like palliative sedation [2,3,4]. This practice, defined as the controlled use of sedatives (e.g., midazolam) to alleviate refractory symptoms [5,6], raises an understudied ethical and clinical dilemma: Can unconscious patients still perceive auditory stimuli, and if so, how should this influence their care?

Emerging evidence challenges traditional assumptions about consciousness during sedation. Neuroimaging studies—including fMRI, EEG, and PET—primarily conducted in anaesthesia and intensive care unit contexts, report persistent brain activity in sedated patients near death, including neural responses to simple auditory stimuli [7]. It is important to note that these findings represent indirect evidence when applied to palliative sedation, as sedation protocols, patient physiology, and clinical goals differ significantly between intensive care unit or anaesthesia settings and end-of-life palliative care. For example, sedation in an intensive care unit is often shorter-term and uses different drug combinations and dosages compared to the prolonged, symptom-focused sedation typical of palliative care. Nonetheless, these neurophysiological findings suggest that (i) the brain may resist hypoxia longer than previously thought, potentially due to midazolam’s neuroprotective properties [7,8]; (ii) subcortical structures such as the thalamus might process emotional stimuli even without conscious awareness [9,10,11]; and (iii) clinical observations —such as the calming effects of familiar voices or music in anesthetized patients—support the potential for non-conscious emotional processing [12,13,14,15,16].

Despite growing awareness of auditory perception at the end of life—and the potential for hearing limitations due to congenital conditions, aging, or treatment-related causes (i.e., chemotherapy, ototoxic antibiotics, or radiotherapy)—current palliative sedation guidelines rarely address auditory care explicitly. While some recommend avoiding distressing stimuli during specific interventions (e.g., death rattle management) [2,17], few offer concrete protocols for communication with sedated patients or their families. Leaves clinicians without evidence-based guidance to answer pressing family questions about their loved ones’ sensory experiences (e.g., “*Can my loved one still hear us*?” or “*Should we avoid certain conversations at the bedside*?”). These gaps reveal three critical ethical imperatives: (i) the moral obligation to curate the acoustic environment for sedated patients, given the possibility of distressing auditory perceptions; (ii) the clinical duty to establish standardized therapeutic communication protocols for patients and families; and (iii) the urgent research priority to study preserved auditory perception and its emotional impact during palliative sedation. Together, these considerations challenge existing palliative care paradigms and call for a revaluation of the end-of-life patient experience.

Therefore, the aim of this review is (i) to evaluate current European palliative sedation guidelines for their recognition of auditory care in unconscious patients, identifying both existing recommendations and critical omissions, and (ii) to develop evidence-based clinical recommendations for integrating auditory care—including hearing preservation and sound environment management—into standardized end-of-life palliative care practice.

## 2. Materials and Methods

This narrative review explores auditory perception during palliative sedation, focusing on (i) hearing preservation in end-of-life care; (ii) sensory processing in the terminal phase; and (iii) clinical practices and recommendations.

An initial literature search was conducted in MEDLINE, Scopus, Cochrane Library, and CINAHL Complete using terms related to palliative sedation, hearing, and sensory perception. However, the retrieved studies primarily addressed general sedation practices or overlooked specific aspects of auditory care, including guidance for family members regarding attention to hearing during the final stages of life.

Given the paucity of primary research on this topic, the review subsequently prioritized targeted searches in authoritative institutional repositories: (i) the European Palliative Sedation Repository (https://palliativeprojects.eu/palliativesedation/ (accessed on 28 March 2025)) and (ii) the European Association for Palliative Care (EAPC) (https://eapcnet.eu/eapc-publications/ (accessed on 28 March 2025)). These sources were selected for their ability to (i) consolidate multidisciplinary expert consensus; (ii) reflect current clinical protocols within European palliative care networks; and (iii) address gaps in the primary literature through practical, experience-based guidance.

## 3. Results

### 3.1. Foundation and Evolution of Palliative Sedation Guidelines

The EAPC established a foundational framework for palliative sedation in 2009, recognizing it as an ethically acceptable last-resort intervention for terminal patients experiencing refractory symptoms [18]. This seminal document represented a crucial step in standardizing sedation practices across Europe. However, both preceding and subsequent guidelines have demonstrated significant variations in their approaches, with differences persisting even within national boundaries. This analysis particularly focuses on aspects related to auditory preservation, maintenance of consciousness, and humanization of care during palliative sedation, examining how these elements are addressed in major guidelines.

### 3.2. Key Guidelines and Recommendations

The EAPC framework [19] serves as the cornerstone for palliative sedation practices in Europe. This guideline advocates for a carefully titrated approach to medication administration, emphasizing the importance of achieving symptom relief while minimizing unnecessary suppression of consciousness. It requires thorough documentation of dosage adjustments and patient responses throughout the sedation process. For consciousness assessment, the guideline recommends monitoring responses to various stimuli, including agitation triggers, motor activity, and facial expressions, with particular attention to maintaining physiological stability during intermittent sedation to allow for potential recovery.

Beyond pharmacological considerations, the EAPC framework places strong emphasis on humanized care practices [19]. It mandates that healthcare teams maintain the same level of dignified treatment throughout the sedation process, including verbal communication with patients and environmental adjustments. The guideline actively encourages family presence, recognizing the therapeutic value of farewell rituals and emotional closure. Specific recommendations guide family members in providing non-medical care, such as speaking to the patient, physical touch, oral care, and creating a personalized atmosphere through music, reading, or spiritual practices. While these elements are not explicitly framed as hearing care, they inherently involve auditory stimulation and environmental sound management. Published in 2009, this comprehensive guideline has served as the blueprint for numerous subsequent adaptations across European healthcare systems.

### 3.3. Comparative Analysis of European Guidelines

A comprehensive review of European guidelines on palliative sedation from eight countries (Belgium, Germany, Hungary, Italy, the Netherlands, Romania, Spain, and the UK) [20] reveals both consistencies and variations in practice. Accordingly, sedation precedes an estimated 10–18% of deaths in Europe [6,21,22,23,24,25,26,27,28,29,30]. Its application is notably influenced by cultural, religious, and social factors, with considerable variability not only between countries and cultures but also among individual institutions [21]. Moreover, these findings highlight a shared ethical emphasis across national guidelines on using sedation proportionally and judiciously [21]. While minimal sedation is generally preferred to relieve suffering, deeper and continuous sedation (a form of palliative sedation used to relieve refractory symptoms in terminal patients [31]) tends to be limited to severe or end-of-life situations with unmanageable distress.

The reviewed guidelines uniformly emphasize the importance of multidisciplinary decision-making and robust family support systems [18]. Pharmacological approaches show remarkable consistency, with midazolam universally recognized as the first-line medication, and levomepromazine widely adopted as the secondary option. For clinical assessment, most guidelines recommend using validated tools such as the Critical-Care Pain Observation Tool (CPOT) for suffering evaluation and various consciousness scales (RASS-PAL, Sedation Rating Scale, Rudkin Scale, or Ramsay Scale) for monitoring sedation depth. Notably, while these guidelines implicitly support auditory participation through recommendations on verbal communication and music, none explicitly address auditory care as a distinct consideration in sedation protocols.

An international survey of palliative sedation practices in these eight countries [20] reinforces these findings while providing additional practical insights. The survey highlights essential care protocols including meticulous oral and eye care, hygiene maintenance, pressure ulcer prevention, and appropriate catheterization. Family involvement emerges as a consistent priority, with recommendations for providing private spaces for emotional expression and farewell rituals. The survey again notes the encouragement of family members to engage in non-medical care activities such as talking to or touching the sedated patient.

Some protocols offer specific practical recommendations for creating an optimal care environment. These include maintaining a calm, private space [20,32,33,34,35] with soft lighting [33,34] to facilitate physical and emotional interaction. Specific protocols offer practical recommendations to create an optimal care environment, including maintaining soft vocal tones [20,33,34] and explaining to families that hearing and touch persist until the end of life [15]. Other guidelines emphasize that patients may still process sensory stimuli even with cognitive impairment [33] and that an absence of response does not necessarily indicate unconsciousness [36].

### 3.4. Global Guidelines and Recent Updates

The analysis of international guidelines [37] reveals both convergence and divergence in palliative sedation practices. Cultural influences manifest particularly in varying definitions of sedation types (continuous versus intermittent) and in thresholds for determining refractoriness of symptoms. A significant challenge identified across guidelines is the lack of standardized terminology, which contributes to inconsistent reporting and implementation of sedation practices worldwide.

Recent updates to the EAPC framework through an international Delphi study [21] and subsequent revision [21] have refined several aspects of palliative sedation while maintaining core principles. Pharmacological recommendations now present a more detailed hierarchy: midazolam remains the first-line agent, with levomepromazine/chlorpromazine as secondary options, lorazepam as an alternative, and propofol reserved for specific cases [21]. These revisions place increased emphasis on mitigating family distress during the sedation process, explicitly recommending maintained verbal and physical contact with patients, personalized environmental adjustments (including music and aromas), and continued oral care. The 2024 Delphi study employed the AGREE II tool to rigorously assess guideline quality [21]. Despite these updates, auditory care continues to receive only implicit attention.

### 3.5. Consensus and Gaps

The analysis reveals strong international consensus on several fundamental principles of palliative sedation [18,19,20,21,37]. Proportional sedation, administered by multidisciplinary teams with patient comfort as the paramount goal, represents the universal standard of care. The use of benzodiazepines as the preferred first-line sedative agent is consistently recommended across international guidelines. Significant divergences arise in the definition of refractory symptoms and adaptation of protocols to accommodate cultural and religious norms.

The most notable gap identified across all reviewed guidelines [18,19,20,21,37] is the lack of explicit attention to hearing care. While multiple guidelines implicitly support auditory engagement through recommendations about verbal communication and music therapy, none formally recognize hearing preservation or auditory stimulation as distinct components of palliative sedation protocols.

### 3.6. Comprehensive Care Recommendations for Palliative Sedation

Based on analysis of international guidelines, we propose these essential care recommendations for palliative sedation:

- Human dignity and individualized care
  Every sedated patient deserves treatment with fundamental respect until dead, receiving fully individualized interventions that combine professional expertise with humanized attention [19,20,21,33]. Healthcare teams should provide comprehensive care focused on alleviating suffering while preserving patient dignity throughout the sedation process [19,20,21,33].
- Communication and family support
  Active communication with both patients and families forms the cornerstone of quality care. This includes (i) maintaining open information channels [34,35]; (ii) practicing active listening for both verbal and nonverbal expressions of needs [33]; (iii) regularly assessing family emotional states [20,21,38]; and (iv) encouraging emotional expression from relatives [20,39].
- Sensory environment optimization
  Creating an appropriate sensory environment requires (i) quiet, private spaces facilitating emotional and physical interaction [19,21,33,35,40]; (ii) soft lighting to reduce sensory overload [33,35]; (iii) conscious use of calm vocal tones [33,35]; and (iv) protection from inappropriate comments or negative stimuli [33].
- Sensory preservation strategies
  Clinical teams should (i) explain to families that hearing and touch persist until death [2,19]; (ii) clarify that unresponsiveness does not necessarily indicate unconsciousness [17]; (iii) encourage meaningful touch and verbal communication even with unresponsive patients [2,35,41]; and (iv) note that patients may process sensory stimuli despite cognitive impairment [34].
- Family engagement guidelines
  Healthcare providers should (i) actively encourage family participation in care [19,35]; (ii) explain the preserved capacity for sensory perception during sedation [17,41]; (iii) guide families in creating peaceful environments with music or familiar voices [17,19,41]; and (iv) support physical contact and reassuring communication [2,39].

**Figure 1:**
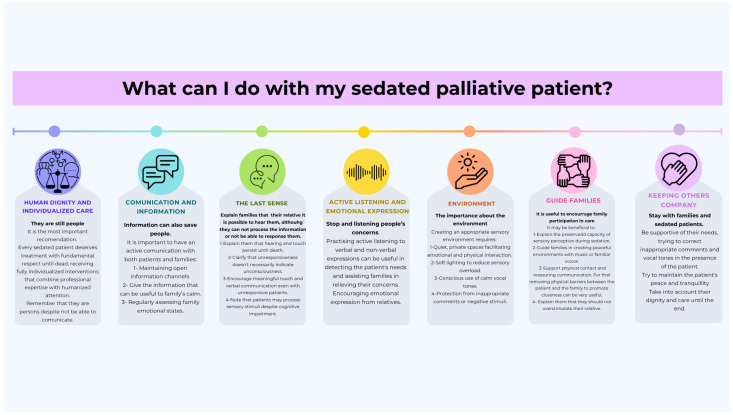
Principles of dignity-centred palliative care: a guide for healthcare professionals [2,19,20,21,33,34,35,38,41].

**Figure 2:**
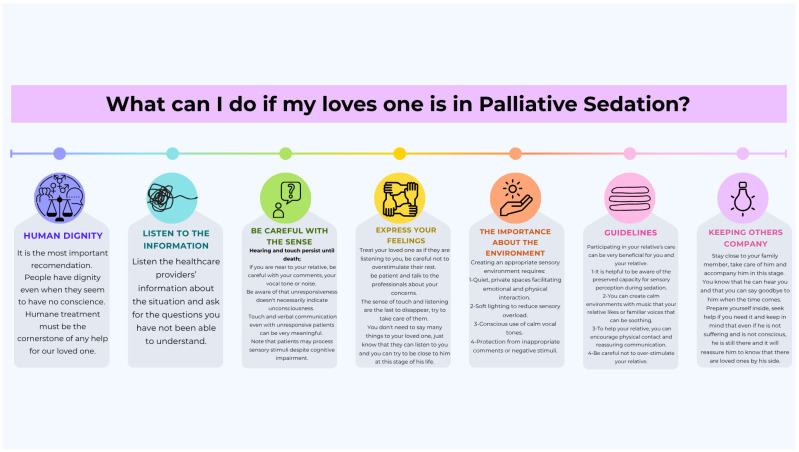
Principles of dignity-centred palliative care: a guide for families [2,17,19,21,33,35,39,40,41].

These recommendations synthesize evidence from guidelines (Figure 1 and Figure 2), providing a comprehensive framework for sensory-aware palliative sedation practice. The explicit focus on hearing preservation and family guidance in Spanish documents [2,17,34,35,41] offers important practical enhancements.

## 4. Discussion

### 4.1. Heterogeneity in European Guidelines and the Leadership of EAPC

The EAPC 2009 framework [19] has become the principal reference for palliative sedation across Europe, forming the basis for most national guidelines. Despite its widespread recognition, a significant gap remains between theoretical awareness and clinical application. Research indicates that while up to 86% of healthcare professionals are familiar with the EAPC guidelines, fewer than 20% consistently implement them in practice [36]. This implementation gap likely stems from two key challenges specific to the European context.

First, substantial cultural and ethical differences shape how palliative sedation is conceptualized across countries. These disparities are particularly evident in the interpretation of “refractory symptoms” and the varying acceptance of deep sedation [18,20,21,37], and they have contributed to divergent approaches in guideline development due to the absence of standardized terminology.

This heterogeneity presents both challenges and opportunities. On the one hand, it allows for culturally sensitive adaptations of palliative care; on the other hand, it leads to inconsistencies in clinical practice, potentially affecting the quality and uniformity of care across or even within countries. In this context, the EAPC’s leadership is crucial, providing a common foundation while acknowledging regional flexibility. However, the low rate of practical implementation suggests that bridging the gap between guideline development and real-world application requires further efforts. Future initiatives should focus on developing adaptable implementation strategies that respect cultural and ethical differences while maintaining the core principles of palliative care.

Despite these variations, several fundamental principles consistently emerge across European guidelines [18,19,20,21]. The primary objective remains clear: to minimize suffering while preserving patient dignity. First, the pharmacological approach shows notable consistency, with midazolam universally recommended as the first-line sedative due to its favourable pharmacokinetics, while levomepromazine is the preferred alternative. Second, all guidelines emphasize a multidisciplinary framework, integrating medical, psychological, and spiritual care teams to address patients’ complex needs. Third, the principle of humanized care is a constant theme, emphasizing the importance of maintaining communication with both the patient (even during sedation) and family members. This includes clear information-sharing, emotional support, and respect for patient preferences throughout the sedation process. These shared elements suggest that despite practical variations, the ethical foundations of palliative sedation remain remarkably consistent across different healthcare systems and cultural contexts.

These shared elements suggest that despite practical variations, the ethical foundations of palliative sedation remain remarkably consistent across different healthcare systems and cultural contexts. This consistency provides an ideal foundation for integrating sensory-aware protocols, particularly regarding auditory preservation, which aligns with these universal principles of humanized care.

### 4.2. Hearing as an Underestimated Dimension in Palliative Sedation

Current palliative sedation guidelines do not explicitly address auditory care, yet several implicit recommendations suggest its potential significance. The EAPC’s 2009 framework highlights the importance of verbal communication with sedated patients and encourages environmental adjustments, such as meaningful auditory stimuli (e.g., music, reading) and active family interaction [19]. More recent revisions, including the 2023 guidelines, acknowledge the psychological distress experienced by families when communication with sedated patients is lost, indirectly supporting the idea that maintained auditory connection could alleviate this burden [21].

Emerging scientific evidence supports this perspective. Neurophysiological studies in intensive care settings [7] demonstrate preserved auditory cortex activity in mildly-to-moderately sedated patients, detectable through auditory evoked potential despite the absence of behavioural responses.

Although it is true that these findings are derived from individuals who are not undergoing palliative sedation, they may still be relevant given the pharmacological similarities of the sedative agents used, the comparable effects on brain function, and the scarcity of experimental studies specifically focused on patients receiving that palliative sedation.

However, although the context differs, these clinical studies further support the relevance of auditory perception, reporting a reduction in agitation among terminal patients exposed to therapeutic music [12,13,14,15,42]. So, this creates a paradox in current practice: while palliative care guidelines universally advocate for comprehensive comfort measures, the auditory dimension–fundamental for human connection and emotional processing–remains underdeveloped in formal protocols.

Therefore, although indirect, the evidence suggests that auditory perception may persist even during sedation, functioning both as a therapeutic tool to enhance patient comfort and as a means to foster connection with family. This oversight in guideline development represents a gap between emerging neuroscientific insights and clinical practice standards in palliative sedation. Integrating structured auditory care approaches could enhance patient-centred outcomes and family support, aligning with the holistic principles that define palliative care.

### 4.3. Practical Implications and Proposed Improvements

Systematically incorporating auditory care into palliative sedation protocols could significantly enhance clinical practice. A key first step would be to develop explicit recommendations within existing assessment frameworks, integrating auditory considerations into clinical scales or care guidelines. This could include monitoring excessive noise levels, maintaining verbal communication when appropriate, and providing meaningful auditory stimuli. Standardizing these practices would ensure consistency while allowing flexibility for individual and cultural preferences.

Targeted training programs for healthcare professionals are essential to translate these recommendations into daily practice. Educational initiatives should focus on two main areas: (i) enhancing therapeutic communication skills tailored to patients under sedation and (ii) fostering competence in recognizing and supporting auditory processing in non-responsive individuals. Such training would empower clinicians to act with greater confidence and intentionality, while preserving appropriate sedation levels.

Importantly, the implementation of these interventions is feasible even in low-resource settings. Many auditory care strategies—such as the use of familiar voices or culturally significant music—require minimal equipment and financial investment. The most pressing need is not technological but educational. Basic training in the neurobiology of hearing during sedation, and in the ethical implications of sensory care, can be delivered through scalable, low-cost formats like printed guides, digital modules, or brief workshops. Integrating these practices into existing workflows does not require major structural change but rather a shift in awareness. Moreover, culturally sensitive adaptation is crucial, protocols should reflect local preferences in music, language, and communication styles. For this reason, the proposed recommendations are globally relevant and adaptable to diverse care contexts.

Equally important is the development of comprehensive family support strategies addressing auditory perception. Providing relatives with clear, evidence-informed information about the possibility of preserved hearing may transform how they relate to sedated loved ones, offering them meaningful ways to remain connected, enhance comfort, and possibly ease anticipatory grief. Practical guidance should include how to communicate in a calm, reassuring tone; select music, messages, or readings with personal significance; and create an emotionally safe and acoustically gentle environment.

At the same time, it is essential to clearly communicate potential risks, especially the possibility of overstimulation or distress in cases of deep or fluctuating sedation. Families should be made aware that although sound may be perceived, excessive or inappropriate auditory input could interfere with the patient’s comfort. To minimize unintended harm, caregivers can be encouraged to use low-volume audio, avoiding sudden changes in tone or intensity; speak slowly and soothingly, limiting the number of simultaneous stimuli (e.g., no talking over music); observe the patient’s physiological cues (e.g., breathing rhythm, facial expression) and reduce stimuli if signs of discomfort arise; limit sessions of auditory input to brief, intentional periods with quiet intervals; and coordinate with clinical staff to align auditory interactions with sedation levels and care goals. The emphasis should remain on respectful, gentle interaction, prioritizing presence over stimulation, and connection over conversation. These practical recommendations can empower families to participate meaningfully in end-of-life care while safeguarding the patient’s well-being.

When implemented with sensitivity and respect, auditory care strategies can offer meaningful emotional support to families and enhance patients’ sensory comfort, promoting a more holistic approach that aligns with the core principles of palliative care. Hearing should be recognized as a potentially preserved sense beyond observable responsiveness and thoughtfully integrated into clinical practice. Through small, sound-filled gestures grounded in humanity, we affirm the dignity of the person, even in the final moments of life.

### 4.4. Limitations and Future Directions

Several limitations must be acknowledged when considering auditory care in palliative sedation. A primary challenge lies in the lack of standardization in defining and measuring sedation across clinical and research contexts. Terminological and methodological inconsistencies hinder the comparison of findings and the synthesis of evidence. Without consensus on concepts such as “light” versus “deep” sedation or standardized metrics for evaluating patient responses, the field struggles to build a cohesive evidence base. Establishing internationally agreed-upon definitions and consolidated measurement tools is thus essential to advance research and clinical practice.

Due to the heterogeneous nature of the included documents, with varying formats and levels of detail, standardized tools for formal quality appraisal were not applied. This diversity hindered the use of uniform evaluation criteria.

Although neuroimaging studies from anaesthesia and intensive care settings suggest the persistence of auditory activity under sedation, these findings are indirect and may not fully translate to palliative sedation scenarios. Differences in pharmacological protocols (e.g., midazolam dosing), therapeutic goals, and patient characteristics underscore the need for caution in generalizing such results. Nonetheless, in the absence of direct data from palliative contexts, this review uses these findings as a hypothetical framework to inform clinical reasoning and guide research priorities. There is a pressing need for studies specifically examining auditory processing during palliative sedation at the end of life.

With respect to auditory interventions such as music therapy and voice-based approaches, much of the current evidence derives from perioperative or other non-palliative care settings. The distinct clinical environments, patient conditions, and therapeutic objectives in these contexts limit the direct applicability of such findings to palliative sedation. Extrapolating results from these studies risks oversimplification and may not capture the unique challenges and needs of patients under end-of-life sedation. Therefore, dedicated randomized controlled trials and observational studies within palliative sedation settings are urgently needed to establish evidence-based practices for auditory care.

Furthermore, robust clinical research on auditory interventions in palliative sedation remains limited. While preliminary studies suggest benefits—such as personalized music therapy reducing agitation—controlled trials are scarce. There is a clear need for research assessing how auditory interventions impact outcomes such as agitation reduction, perceived comfort, and family satisfaction. Future studies should employ standardized assessment tools and control for potential confounding variables to generate high-quality, generalizable evidence.

Despite the apparent low cost and simplicity of some auditory interventions, there is a notable absence of data on their cost-effectiveness. Comprehensive economic evaluations are necessary to support broader implementation, particularly in resource-limited settings. Future research should also validate auditory care protocols across diverse clinical environments to ensure their feasibility, efficacy, and cultural adaptability.

Key research priorities include investigating subliminal auditory processing across varying levels of sedation using neuroimaging techniques and controlled auditory stimulus paradigms. In parallel, the development of practical, evidence-based guidelines for sensory care in palliative settings is essential. These guidelines should be culturally sensitive, accounting for variations in sound preferences, communication practices, and end-of-life traditions to ensure global applicability. It is important to note that this review focuses exclusively on European guidelines and contexts, reflecting the shared principles, legislation, and healthcare frameworks within this geographic area. Future research should expand to include diverse perspectives from other continents or regions with similar characteristics to enrich understanding, identify common principles, and develop culturally sensitive auditory care guidelines applicable across different palliative sedation settings worldwide.

This line of inquiry also raises important philosophical and ethical considerations. The possibility that auditory perception persists during palliative sedation reinforces the relevance of sensory care as an expression of human dignity. Our findings support an emerging evidence-based framework for humane end-of-life care—one that integrates contemporary neuroscientific knowledge with dignity-preserving practices. This dual approach bridges the gap between biological plausibility and clinical protocol, promoting models of care that recognize potential residual perception while ensuring patient and family-centred support.

Future research must continue to bridge neuroscience and palliative ethics through two complementary approaches: (i) rigorous clinical studies to quantify sensory preservation across sedation levels and (ii) phenomenological investigations aimed at refining sensory-inclusive care frameworks. Such work will help transform the current hypothesis of perceptual preservation into an established pillar of palliative care. Future research should include specific studies such as EEG monitoring of sedated patients exposed to speech or music, qualitative interviews with family members, and randomized controlled trials evaluating the effectiveness of auditory interventions like music therapy. These targeted approaches will help build a stronger evidence base to guide clinical practice in palliative sedation.

Looking ahead, this article seeks to inspire the development of practical guidance for palliative care services, offering recommendations based on current hearing-related evidence in palliative sedation. This includes promoting multidisciplinary, integrative care models and creating educational programs for families to foster awareness and participation in compassionate, perception-aware end-of-life practices.

## 5. Conclusions

Current evidence suggests that auditory perception may persist during palliative sedation, potentially allowing emotional processing despite lack of visible responses. Our findings advocate for a paradigm shift toward sensory-informed palliative care, where clinical protocols acknowledge that human perception transcends visible responses in altered consciousness states. While conclusive proof is still needed, the precautionary principle compels us to acknowledge this sensory capacity in clinical practice. Notably, existing guidelines have largely overlooked auditory preservation—a significant gap in comprehensive palliative care. Addressing this shortcoming would enhance patient-centered care and provide meaningful support for families, ultimately creating care systems that fully honour the perceptual experiences of dying patients while supporting those who accompany them in life’s final journey.

## Data Availability

Not applicable.
